# Apoptosis-related deregulation of proteolytic activities and high serum levels of circulating nucleosomes and DNA in blood correlate with breast cancer progression

**DOI:** 10.1186/1471-2407-11-4

**Published:** 2011-01-06

**Authors:** Carina Roth, Klaus Pantel, Volkmar Müller, Brigitte Rack, Sabine Kasimir-Bauer, Wolfgang Janni, Heidi Schwarzenbach

**Affiliations:** 1 Institute of Tumor Biology, University Medical Center Hamburg-Eppendorf, Germany; 2 Clinic of Gynecology, University Medical Center Hamburg-Eppendorf, Hamburg, Germany; 3 First Department of Obstetrics and Gynecology, Ludwig Maximilians University of Munich, Munich, Germany; 4 Department of Gynecology and Obstetrics, University of Duisburg-Essen, Essen, Germany; 5 Clinic of Gynecology, Heinrich Heine University, Düsseldorf, Germany

## Abstract

**Background:**

As cell-free circulating DNA exists predominantly as mono- and oligonucleosomes, the focus of the current study was to examine the interplay of circulating nucleosomes, DNA, proteases and caspases in blood of patients with benign and malignant breast diseases.

**Methods:**

The concentrations of cell-free DNA and nucleosomes as well as the protease and caspase activities were measured in serum of patients with benign breast disease (n = 20), primary breast cancer (M0, n = 31), metastatic breast cancer (M1, n = 32), and healthy individuals (n = 28) by PicoGreen, Cell Death Detection ELISA, Protease Fluorescent Detection Kit and Caspase-Glo^®^3/7 Assay, respectively.

**Results:**

Patients with benign and malignant tumors had significantly higher levels of circulating nucleic acids in their blood than healthy individuals (p = 0.001, p = 0.0001), whereas these levels could not discriminate between benign and malignant lesions. Our analyses of all serum samples revealed significant correlations of circulating nucleosome with DNA concentrations (p = 0.001), nucleosome concentrations with caspase activities (p = 0.008), and caspase with protease activities (p = 0.0001). High serum levels of protease and caspase activities associated with advanced tumor stages (p = 0.009). Patients with lymph node-positive breast cancer had significantly higher nucleosome levels in their blood than node-negative patients (p = 0.004). The presence of distant metastases associated with a significant increase in serum nucleosome (p = 0.01) and DNA levels (p = 0.04), and protease activities (p = 0.008).

**Conclusion:**

Our findings demonstrate that high circulating nucleic acid concentrations in blood are no indicators of a malignant breast tumor. However, the observed changes in apoptosis-related deregulation of proteolytic activities along with the elevated serum levels of nucleosomes and DNA in blood are linked to breast cancer progression.

## Background

Every year, more than one million women are worldwide diagnosed with breast cancer, the second most common tumor entity after lung cancer. At the time of initial diagnosis approximately 5% of patients are found to have advanced or metastatic disease [1]. Although screening techniques, surgical and radiotherapy interventions of breast cancer patients have improved the curative rate, a certain percentage of women will develop recurrent or metastatic disease after adjuvant treatment [2]. Therefore, the development of a preoperative blood test that is able to detect early breast tumors and determine regional lymph node or distant metastasis would be desirable.

The quantification of cell-free DNA, which circulates in high concentrations in blood of patients with various malign and benign lesions [3], could support such a non-invasive test. The mechanism of DNA release into the blood circulation occurs either by proliferating or dying cells, such as apoptotic and necrotic cells [4]. It is supposed that this DNA circulates preferentially as mono- and oligonucleosomes. As a late event of apoptosis, intracellular endonucleases, such as caspase-activated DNases, are induced and cleave chromatin at the easily accessible internucleosomal linker regions into mono- or oligonucleosomes [5]. Dying cells are usually phagocytosed by macrophages, but an excess of cell death leads to saturation of this process and to elevated levels of fragmented nucleosomal DNA in the blood circulation [6]. As nucleosomes are rapidly degraded and hydrolyzed from the blood circulation by DNases and proteases, their half life in blood seems to be short [7].

The elevated levels of circulating DNA in blood of cancer patients have been shown to reflect the characteristics of tumor DNA, and to harbor genetic and epigenetic alterations [3]. Previously, we demonstrated the potential of circulating tumor-associated DNA in blood for molecular diagnosis and its prognostic value to identify breast cancer patients at high risk for relapse [8-11]. Accordingly, circulating nucleosomes in blood may also provide an indication of tumor DNA. Although numerous studies focused on the quantification of circulating DNA, only some studies have analyzed the clinical relevance of circulating nucleosomes for diagnosis and prognosis [12-16]. As compared with healthy controls increased concentrations of circulating nucleosomes were measured in various tumor entities [12], but only two studies dealt on breast cancer [17,18].

An essential aspect for cancer development and progression is the deregulation of caspases involved in the regulated apoptotic cascade which consequently, leads to the release of DNA or nucleosomes into the blood circulation [19]. Caspases are cysteine proteases belonging to a large family which constitutes of serine, aspartic and metallo proteases. Proteases may promote cancer progression, because they degrade the extracellular matrix and facilitate subsequent invasion of tumor cells into the surrounding normal tissue. As a result of the aberrantly activated signal transduction in tumors, these enzymes are frequently overexpressed in various cancer entities. The prevailing acceptance that proteases are involved in metastasis led to the development of small-molecule inhibitors for the treatment of cancer [20].

The aim of the present study was to investigate the influence of protease and caspase activities on the levels of circulating nucleosomes and DNA in blood of breast cancer patients, and to correlate the obtained data with the clinicopathological parameters of these patients. Despite the small sample sizes of the cohorts, the broad range of preliminary results shows that the quantification of these determinants could be used as a rapid, non-invasive and blood-based screening tool for the early detection of breast tumor progression.

## Methods

### Tumor patients and healthy volunteers

The present study was conducted at the Clinic of Gynecology of the University Medical Center Hamburg-Eppendorf, at the Ludwig Maximilians University of Munich and at the Department of Gynecology and Obstetrics of the University Hospital of Essen. Between April 2006 and September 2008 blood serum was obtained from 20 patients with benign breast disease (University Hospital of Essen). During January 2008 to August 2009, blood serum was taken from 31 patients with primary breast cancer (M0) after surgery before initiation of adjuvant therapy (Ludwig Maximilians University of Munich). During January to April 2009, blood serum from 32 patients with metastatic breast cancer (M1) was collected 1 to 13 years after surgery of the primary tumor (University Medical Center Hamburg-Eppendorf). Additionally, 28 female controls aged between 21 and 67 with no history of cancer were recruited. The median ages of patients with benign and malignant breast disease was 49 (range 18-79) and 56 (range 34-82), respectively. The data of the tumor patients and healthy controls are summarized in Table 1.

**Table 1:** Patient characteristics and their correlations with the nucleic acid levels and protease/caspase activities in blood

	Patients (%)	Nucleosome levels	DNA levels	Protease activities	Caspase activities
		**Mean values ± Standard Deviation** **Median** **Percentiles (10^th^, 90^th^)**			
		
**Breast Cancer Patients**					
**Total**	63				
**Age**	56 years (range 34 - 82 years)				
**Distant metastasis**					
^**£**^**M0**	31 (49.2)	^**1 **^**878 ± 123** 851 767, 1078	^**2 **^**8241 ± 701** 8293 7232, 9195	^**3 **^**22218 ± 12236** 12682 10401, 35242	35895 ± 62242 7990 2550, 134240
^**$**^**M1**	32 (50.8)	^**1 **^**894 ± 112** 841 812, 1076	^**2 **^**9933 ± 4430** 8756 7237, 16836	^**3 **^**32337 ± 9021** 33326 13573, 45296	11486 ± 9167 8570 4718, 31628
^**#**^**Tumor stage**					
**pT1-2**	29 (53.7)	866 ± 31 858 822, 1058	8352 ± 728 8528 6559, 10246	^**4 **^**11042 ± 750** 10966 10412, 41664	^**5 **^**5386 ± 2042** 5545 2655, 24540
**pT3-4**	25 (46.3)	829 ± 125 769 763, 1079	7690 ± 389 7564 7240, 19519	^**4 **^**30439 ± 10052** 34378 12258, 42790	^**5 **^**66341 ± 93967** 26670 2736, 181806
^**#**^**Lymph node metastasis**					
**N0**	13 (23.6)	^**6 **^**814 ± 80** 769 767, -	8027 ± 518 8011 7517, -	26649 ± 14005 34216 10488, -	19370 ± 17547 12350 6420, -
**N1-3**	42 (76.4)	^**6 **^**860 ± 85** 844 781, 1079	8079 ± 737 8149 6901, 10904	17366 ± 10972 11167 10408, 43572	34821 ± 74658 6890 2613, 66132
^**#**^**Grading**					
**I-II**	25 (46.3)	813 ± 39 827 762, 1057	7914 ± 896 7885 6807, 9056	18737 ± 11916 11964 10001, 37741	47295 ± 99075 7570 2544, 225815
**III**	29 (53.7)	878 ± 98 878 768, 1118	8183 ± 494 8137 6984, 15827	19819 ± 12451 11165 10469, 39564	19671 ± 27313 6655 2907, 51685
^**#**^**Estrogen receptor**					
**positive**	41 (68.3)	846 ± 92 837 767, 1078	7924 ± 719 7868 7185, 9298	20468 ± 12204 11964 10356, 45976	38382 ± 77766 7570 2547, 97376
**negative**	19 (31.7)	861 ± 65 885 767, -	8428 ± 445 8351 6357, -	16574 ± 11766 10827 10423, -	14330 ± 16701 6655 4670, -
^**#**^**Progesterone receptor**					
**positive**	36 (60)	825 ± 44 830 768, 1010	7927 ± 796 8124 6949, 9039	17617 ± 11811 11163 10135, 46261	7317 ± 4533 7152 2532, 25178
**negative**	24 (40)	875 ± 107 865 764, 1144	8209 ± 561 8149 6805, 18757	21094 ± 12382 12682 10456, 35961	55703 ± 90000 6890 2910, 164920
^**#**^**HER2**					
**positive**	23 (39.7)	855 ± 88 844 767, 1078	8148 ± 628 8149 7249, 9183	19548 ± 11973 11167 10312, 39775	35294 ± 74482 6890 2544, 114262
**negative**	35 (60.3)	832 ± 70 823 772, 1193	7774 ± 919 8011 6397, 24618	18650 ± 13486 11245 10564, 45011	17637 ± 18799 7150 4765, 38669

**Benign Breast Tumor Patients**					
**Total**	20				
**Age**	49 years (range 18 - 79 years)				
		831 ± 102 794 768, 922	9306 ± 1156 8837 7611, 11240	16837 ± 5295 14845 14697, 30293	5580 ± 4186 4610 2714, 9307
**Leukocytes**					
**<10.7/nl**	16 (80)	808 ± 61 788 768, -	9471 ± 1068 9191 8496, -	18044 ± 6780 15016 14689, -	4957 ± 2485 4550 2710, -
**>10.7/nl**	1 (5)	1228 - -, -	11626 - -, -	- - -, -	3010 - -, -
**CRP**					
**<0.5 mg/dl**	11 (55)	780 ± 16 780 768, -	9191 ± 235 9191 9024, -	24387 ± 10441 24387 17004, -	5835 ± 4363 5835 2750, -
**>0.5 mg/dl**	5 (25)	823 ± 72 795 772, -	9611 ± 1343 9281 8496, -	14873 ± 197 14831 14689, -	4518 ± 1782 4550 2710, -
**CEA**					
**<3.5 ng/ml**	15 (75)	815 ± 65 791 768, -	9383 ± 1169 9024 8496, -	18715 ± 7354 15141 14771, -	5292 ± 2622 5820 2710, -
**>3.5 ng/ml**	1 (5)	772 - -, -	9913 - -, -	14689 - -, -	3280 - -, -
**CA-125**					
**<65 U/ml**	15 (75)	808 ± 61 788 768, -	9471 ± 1068 9191 8496, -	18044 ± 6780 15016 14689, -	4957 ± 2485 4550 2710, -
**>65 U/ml**	2 (10)	1011 ± 307 - -, -	9926 ± 2404 - -, -	14799 - -, -	3735 ± 1025 - -, -

**Healthy Women**					
**Total**	28				
**Age**	41 years (range 21 - 67 years)				
		726 ± 50 725 715, 809	7747 ± 678 7641 7175, 8801	21133 ± 8028 14996 13166, 30500	5182 ± 2379 5075 3064, 9277

^£^M0, patients with localized breast cancer

^**$**^M1, patients with metastatic breast cancer

^#^parameters of M0 patients

^1^p = 0.01, ^2^p = 0.04, ^3^p = 0.008, ^4^*p = 0.009, ^5^p = 0.009, ^6^p = 0.004

p values as determined by Mann and Whitney-U test (in bold)

*p values as determined by T test (in bold)

Informed written consent was obtained from all diseased and healthy individuals, and the study was approved by the Local Research Ethics Committee.

### Quantification of circulating cell-free DNA and nucleosomes in blood serum

For quantification of the relative amounts of cell-free DNA and nucleosomes in serum, the Quant-iT PicoGreen dsDNA Kit (Invitrogen, Karlsruhe, Germany) and the Cell Death Detection ELISA plus kit (Roche Diagnostics, Mannheim, Germany) were performed, respectively, and carried out according to the manufacturer's protocol.

### Quantification of the protease activity in blood serum

For quantification of the relative protease activity, the Protease Fluorescent Detection Kit (Sigma-Aldrich, Taufkirchen, Germany) was used, and carried out according to the manufacturer's protocol. This kit is based on the proteolytical hydrolysis of a Fluorescein Isothiocyanat (FITC)-labeled casein-substrate detecting all four proteases (serine, aspartic, cysteine and metallo proteases). A three-point standard curve of 1:10, 1:20 and 1:40 dilutions of Trypsin Control was prepared.

### Quantification of the activities of caspases 3 and 7 in blood serum

For measurements of the activities of caspases 3 and 7, the Caspase-Glo^®^3/7 Assay (Promega, Mannheim, Germany) was carried out according to the manufacturer's instructions. This kit is based on the cleavage of the DEVD sequence of a luminogenic substrate by the caspases 3 and 7 resulting in a luminescent signal.

### Statistical analyses

The statistical analyses were performed using the SPSS software package, version 18.0 (SPSS Inc. Chicago, IL). The chi Square or two-tailed Fischer's exact test was used to identify possible associations of the measured parameters. For non parametric comparisons, univariate analyses of the Mann Whitney-U test and the T test of two independent variables, and bivariate analyses of the Spearman's rank correlation were performed. Diagnostic power of the single markers was analysed by receiver operating characteristic (ROC) curves. Areas under the curves (AUC) were calculated. A p-value ≤ 0.05 was considered as statistically significant. All p-values are two-sided.

## Results

### Quantification of circulating nucleosomes and DNA in serum

**Figure 1:**
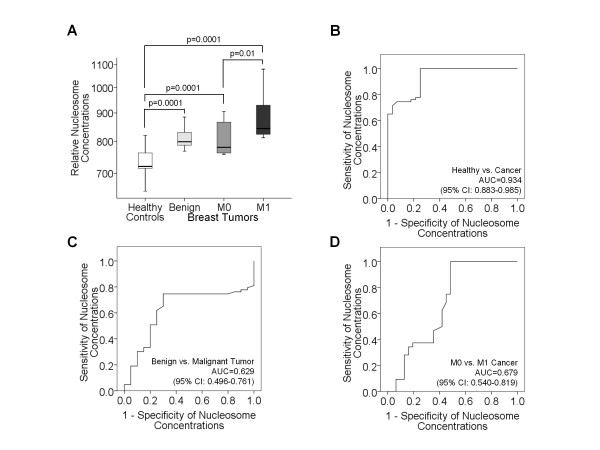
**Comparative analyses of circulating nucleosomes in blood of tumor patients and healthy individuals**. The box plots compare the circulating nucleosome concentrations of healthy individuals (n = 28) with those of patients with benign breast disease (n = 20), primary breast cancer (M0, n = 31) and metastatic breast cancer (M1, n = 32) (**A**). The statistical significances of these comparative analyses, as determined by the Mann and Whitney-U test for the non parametric comparison of two independent variables, are indicated. The ROC analyses show the profiles of sensitivity and specificity of nucleosome concentrations to discriminate healthy controls from breast cancer patients (**B**), benign tumor patients from breast cancer patients (**C**) and M0 from M1 breast cancer patients (**D**).

**Figure 2:**
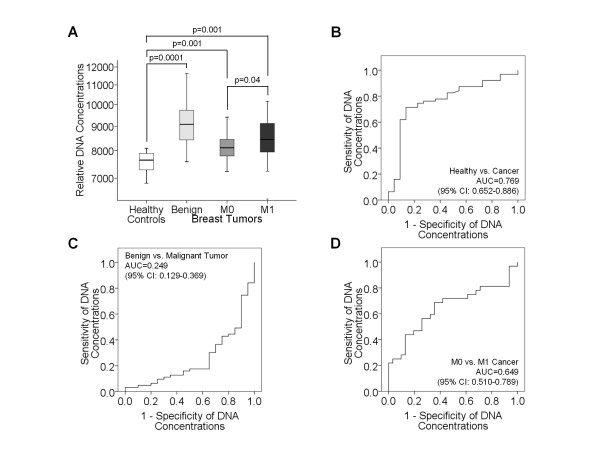
**Comparative analyses of cell-free DNA in blood of tumor patients and healthy individuals**. The box plots compare the circulating DNA concentrations of healthy individuals (n = 28) with those of patients with benign breast disease (n = 20), primary breast cancer (M0, n = 31) and metastatic breast cancer (M1, n = 32) (**A**). The statistical significances as determined by the Mann and Whitney-U test are indicated. The ROC analyses show the profiles of sensitivity and specificity of DNA concentrations to discriminate healthy controls from breast cancer patients (**B**), benign tumor patients from breast cancer patients (**C**) and M0 from M1 breast cancer patients (**D**).

We compared the levels of circulating nucleosomes (Figure 1) and DNA (Figure 2) in blood serum of 28 healthy individuals, 20 patients with benign breast disease, 31 primary breast cancer (M0) and 32 metastatic breast cancer (M1) patients.

As shown in the box plots of Figure 1A, healthy individuals displayed significantly lower nucleosome levels in their blood than patients with benign and malignant breast lesions (p = 0.0001). As determined by univariate analyses of the Mann Whitney-U test, the nucleosome concentrations in blood of M1 patients were higher than those of patients with benign disease and M0 patients (p = 0.01, Table 1) who had similar concentrations (Figure 1A). In order to determine the sensitivity and specificity of the circulating nucleosomes in distinguishing healthy controls from breast cancer patients (Figure 1B), benign tumor patients from breast cancer patients (Figure 1C) and M0 from M1 breast cancer patients (Figure 1D), we performed ROC analyses and used area under the ROC curve (AUC) to test its performance. The AUC values were between 0.629 and 0.934, demonstrating the significant differences of the nucleosome levels (Figure 1).

As shown in the box plots of Figure 2A, healthy individuals also displayed significantly lower DNA concentrations in their blood than patients with benign (p = 0.0001) and malignant (p = 0.001) breast lesions. Surprisingly, high serum levels of DNA could be detected in patients with benign breast disease. Patients with benign breast tumor had the highest levels, and M1 patients had higher levels than M0 patients (p = 0.04, Table 1). In order to determine the sensitivity and specificity of the cell-free DNA in distinguishing healthy controls from breast cancer patients (Figure 2B), benign tumor patients from breast cancer patients (Figure 2C) and M0 from M1 breast cancer patients (Figure 2D), we performed ROC analyses. The AUC values were between 0.249 and 0.769 (Figure 2).

**Figure 3:**
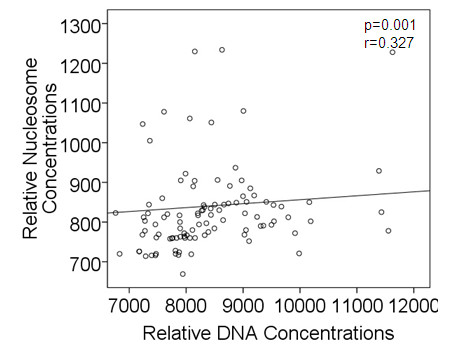
**Significant association between nucleosome and DNA concentrations**. The scatter plot of relative nucleosome vs. DNA concentrations of serum samples from all patients and healthy individuals analyzed shows the distribution of most points on the left corner of the diagram. The relationship of the both variables was significant (Spearman rank test).

As shown in a scatter plot of Figure 3, the bivariate statistical analysis of the serum samples of all patients and healthy individuals revealed a significant correlation between nucleosome and DNA concentrations (p = 0.001, r = 0.327; Spearman rank test).

### Quantification of protease and caspase activities in blood serum

**Figure 4:**
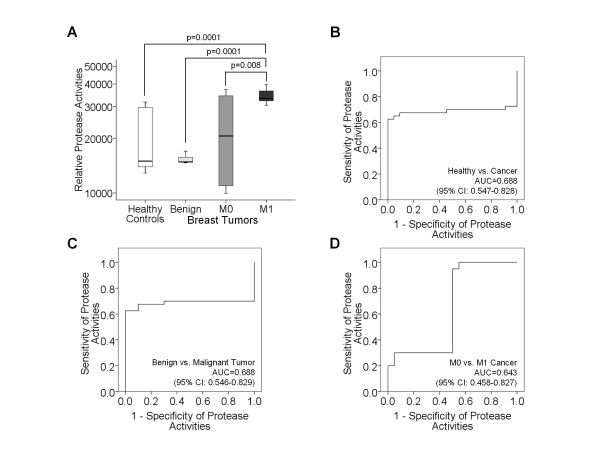
**Comparative analyses of circulating protease activities in blood of tumor patients and healthy individuals**. The box plots compare the relative protease activities in blood of healthy individuals (n = 28) with those of patients with benign breast disease (n = 20), primary breast cancer (M0, n = 31) and metastatic breast cancer (M1, n = 32) (**A**). The statistical significances as determined by the Mann and Whitney-U test are indicated. The ROC analyses show the profiles of sensitivity and specificity of protease activities to discriminate healthy controls from breast cancer patients (**B**), benign tumor patients from breast cancer patients (**C**) and M0 from M1 breast cancer patients (**D**).

**Figure 5:**
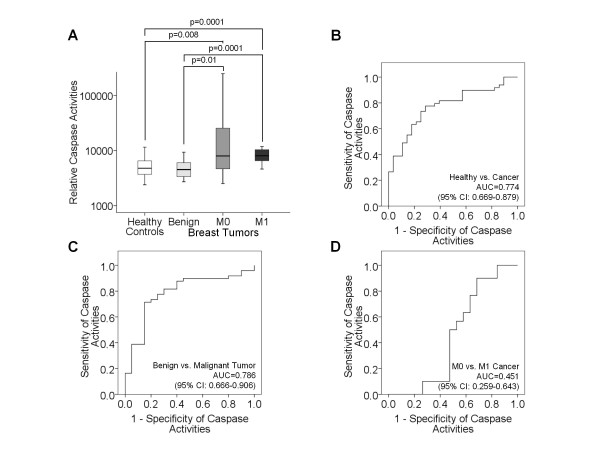
**Comparative analyses of circulating caspase activities in blood of tumor patients and healthy individuals**. The box plots compare the relative caspase activities in blood of healthy individuals (n = 28) with those of patients with benign breast disease (n = 20), primary breast cancer (M0, n = 31) and metastatic breast cancer (M1, n = 32) (**A**). The statistical significances as determined by the Mann and Whitney-U test are indicated. The ROC analyses show the profiles of sensitivity and specificity of caspase activities to discriminate healthy controls from breast cancer patients (**B**), benign tumor patients from breast cancer patients (**C**) and M0 from M1 breast cancer patients (**D**).

To determine the impact of protease and caspase activities on the levels of circulating nucleosomes and DNA, we measured these activities in blood serum of the patient subgroups and healthy individuals (Figures 4 and 5). The protease assay was optimized for measuring the activities of serine, cysteine, metallo and aspartic proteases (Figure 4). In addition, we also separately measured the activities of caspases 3 and 7 (Figure 5), because they play key effecter roles in apoptosis leading to the release of nucleosomes or DNA into the blood circulation.

In contrast to benign breast tumor and M1 patients, a broad range of protease activities was observed in healthy individuals and M0 patients (Figure 4A). In M1 patients the median levels of serum protease activities was approximately 2-2.5-fold higher than in the other subgroups. In order to determine the sensitivity and specificity of the relative protease activities in distinguishing healthy controls from breast cancer patients (Figure 4B), benign tumor patients from breast cancer patients (Figure 4C) and M0 from M1 breast cancer patients (Figure 4D), we performed ROC analyses. The AUC values were >0.642 (Figure 4).

As shown in Figure 5A, both subgroups M0 and M1 had 1.7-fold higher median levels of serum caspase activities than healthy controls and patients with benign breast disease. The AUC values of ROC analyses were between 0.451 and 0.786 (Figure 5B-D).

**Figure 6:**
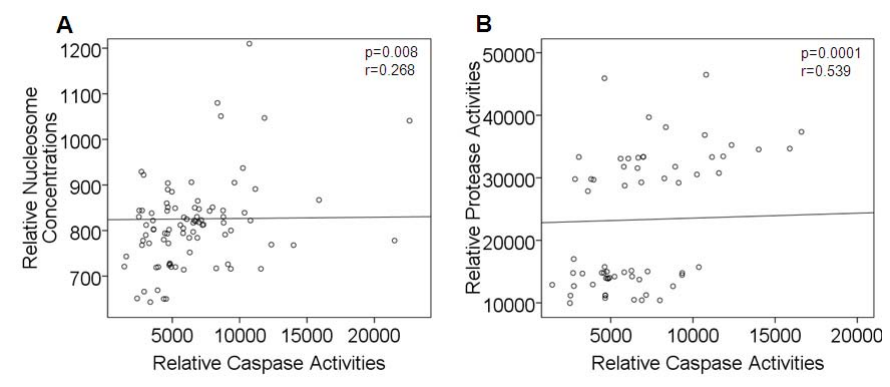
**Significant association between nucleosome, caspase and protease activities**. The scatter plots of relative nucleosome concentrations vs. caspase activities **(A) **and protease activities vs. caspase activities (**B**) show the distribution of the points derived from the serum samples. The relationships of both variables were significant (Spearman rank test).

The Spearman rank test of all analyzed blood samples revealed significant correlations between the circulating nucleosome levels and caspase activities (p = 0.008, r = 0.268; Figure 6A), and between the protease and caspase activities (p = 0.0001, r = 0.539; Figure 6B).

### Clinical relevance of circulating nucleosomes, DNA, proteases and caspases in blood serum

Statistical evaluations of the circulating nucleosome and DNA concentrations as well as protease and caspase activities in blood of 31 M0 and 32 M1 patients were performed with their clinical and histopathological data (Table 1). Significant associations of the elevated serum protease and caspase activities with advanced tumor stages could be observed in M0 patients (p = 0.009). Eighty-five % of women with pT1-2 and 72% of women with pT3-4 had low and high protease activity levels, respectively. Moreover, 82% of patients with pT1-2 and 55% of patients with pT3-4 had low and high caspase levels, respectively. In addition, upregulated protease activities correlated with distant metastases of the cancer patients (p = 0.008). Increasing levels of circulating nucleosomes associated with lymph node metastases (p = 0.004) and distant metastases (p = 0.01), whereas circulating serum DNA associated with the presence of overt metastases in the breast cancer patients (p = 0.04, Table 1).

Statistical evaluations of the circulating nucleosome and DNA concentrations as well as protease and caspase activities in blood of benign breast tumor patients could not be performed with the lab parameters of these patients, because this patient cohort (n = 20) was too small and the distribution of patients within the populations with high and low values was too heterogeneous (Table 1). Of the 20 patients there was only one patient who had simultaneously very pronounced leukocyte, CRP (C-reactive protein) and CA125 values. This patient had also high nucleosome and DNA values.

## Discussion

In the current study we examined the relationship of the serum levels of circulating nucleic acids with the activities of proteases and caspases. In addition, we explored the interplay and the changes in release of these determinants in the blood circulation of breast cancer patients, patients with benign breast disease and healthy individuals.

Although patients with benign and malignant breast tumors had significantly higher levels of circulating nucleosomes and DNA in their blood than healthy individuals, these elevated yields could not discriminate between benign and malignant lesions as documented by our present study. Thus, cell-free nucleic acids may be a molecular biomarker for detecting both, benign and malignant breast diseases in contrast to the circulating protease and caspase activities which were specifically increased in blood of breast cancer patients.

Cell-free DNA may circulate in human blood in diverse forms, as naked DNA, associated with histones as nucleosomes, bound to other plasma proteins or packed in apoptotic bodies [12]. Our detection of the significant association of nucleosome concentrations with DNA concentrations in blood of all patients and healthy individuals emphasized that DNA may predominantly circulate in form of nucleosomes in human blood. A major mechanism underlying the release of nucleosomes into the blood circulation is the internucleosomal cleavage of DNA in small mono- and oligonucleosomal fragments by activated endonucleases. In this apoptotic process caspases play a central role inducing nucleosomal DNA fragmentation by activating DNases. In malignant tumors the apoptotic cell death seems to be increased [21]. As shown by our current findings, the activities of caspases 3 and 7 were significantly upregulated in blood of breast cancer patients in comparison to patients with benign breast disease and healthy individuals, and these increased activities were associated with advanced tumor stages. In particular, our data suggest that in breast cancer patients caspases 3 and 7 are involved in apoptosis which may lead to the excess of circulating nucleosomes in blood of these patients. Additionally, our detection of the upregulation of caspases 3 and 7 in advanced tumor stages may reflect their active role in tumor progression. As demonstrated by immunohistochemical analyses in another study, the caspases 6 and 8, besides the caspase 3, may also be engaged in the apoptosis of breast carcinomas as well as preneoplastic lesions. The expression of these caspases, which was strongly associated with an increased apoptotic index, was significantly lower in benign epithelial hyperplasias than in situ carcinomas and invasive carcinomas [22]. Accordingly, we found no increase in the activities of caspases 3 and 7 in the blood serum of patients with benign disease, but an association of their activities with the concentrations of circulating nucleosomes in all patients. This indicates that apart from caspases 3 and 7, other caspases may likely affect the high levels of nucleic acids in blood of patients with benign breast disease. In contrast to our data, Deligezer et al. obtained no correlation of nucleosomal DNA fragmentation with the occurrence of caspases in patients with lymphoma and myeloma. The reason of this discrepancy is that they did not analyze the activities of the circulating serum caspases 3 and 7, but only the yields of caspase-3 transcripts in lymphocytes [23].

Besides the elevation of caspase activities, we also observed a significant upregulation of protease activities, including the various serine, cysteine, metallo and aspartic proteases, in blood of patients with advanced tumor stages and metastatic disease. Our findings substantiate the active role of proteases in tumor progression and metastases. The degradation of extracellular matrix represents a key event in the multistage process of tumor invasion and metastasis. Such a degradation requires the concerted action of proteases which are involved in the proteolysis of many different components of the extracellular matrix [24]. The cleavage of extracellular matrix proteins has also been related to tissue remodeling and neo-vascularization [25]. These processes support the dissemination of tumor cells into the blood circulation [26,27]. The apoptosis of these cells may also contribute to the elevated levels of nucleic acids in blood [11,28]. In contrast to the high protease activities in breast cancer patients, we found that these activities in blood of patients with benign disease were not increased, endorsing that proteases, as well as the caspases 3 and 7, seem to play a smaller role in benign than in malignant lesions. In particular, the matrix metalloproteinases (MMPs) and tissue inhibitors of metalloproteinases (TIMPs) are thought to be involved in tumor progression and metastasis [29,30]. Consistent to our results on the protease activities, Wu et al. found that serum levels of MMP-9 and TIMP-1 were significantly higher in breast cancer patients than in benign breast disease and healthy controls, and that they associated with tumor progression [31].

Our data suggest that the measurement of circulating nucleosome concentrations may be used for the risk assessment of breast cancer patients who develop metastases, because the elevated nucleosome levels significantly associated with regional and distant metastatic breast disease spreading. However, to sustain the prognostic relevance and the clinical utility of serum nucleosomes, as well as DNA, long-term follow-up analyses have to be performed.

Although we found significantly higher nucleosome levels in blood of lymph node-positive and metastatic breast cancer patients than in patients without overt metastases or with benign breast disease, serum DNA yields were lower in cancer patients than in patients with benign breast disease. However, we detected a significant association between the DNA and nucleosome values of all patients and healthy individuals. This ostensible discrepancy can be explained by the dramatic increase in the nucleic acids in blood of patients with benign disease. Nevertheless, the rise in nucleosome and DNA levels leading from healthy individuals over M0 patients to M1 patients was similar, albeit the inclination of the DNA levels of these three cohorts was somewhat more gently than that of the nucleosome levels. Why the DNA and nucleosome concentrations are so high in our cohort of patients with benign disease is unknown. It is well-known that an increased amount of circulating DNA is not only related to cancer, but is also found in blood of patients with severe inflammatory processes [32]. Since also carcinomas are associated with inflammatory processes, these high DNA levels in patients with benign breast disease are anyhow surprising. However, a previous study showed results alike to ours, indicating that serum levels of circulating cell-free DNA could have diagnostic value to discriminate between healthy individuals and patients with breast lesions, but they have no relevance to discriminate between patients with malignant and benign breast lesions [33]. In addition, Holdenrieder et al. also detected elevated serum levels of nucleosomes in various benign diseases, particularly in infectious diseases, and showed that the quantification of nucleosomes limits the diagnostic capacity for cancer detection [12]. Nucleic acids may be released into the blood circulation by damaged cells which are present in both, benign and malignant diseases. A factor for these high serum concentrations in patients with benign breast tumors could be the delayed clearance of nucleic acids. We tried to determine the DNase activity in the different cohorts, but the measured values were too low to evaluate them. Therefore, we do not know whether the DNA concentrations inversely correlate with DNase levels. However, that DNA is an remarkably robust molecule and resistant against degradation was demonstrated in mice in whom orally ingested DNA survived the digestion in the gastrointestinal tract [34].

In breast cancer the serum tumor markers cancer antigen (CA) 15-3 and carcinoembryonic antigen (CEA) play a role in tumor growth and spreading. Particularly in advanced stages of cancer patients these markers have been shown to be elevated [35]. While in patients with primary breast cancer the concentration of CA 15-3 and CEA are usually within the normal range, increased levels are often observed in patients with metastatic disease, and correlate with increased metastatic load [36]. Moreover, it has been reported that serum nucleosomes showed a significant positive correlation with CA 15-3 in breast cancer patients [37]. Therefore, it would be interesting to investigate whether our data on nucleic acid concentrations and proteolytic activities refer to these protein markers. Since in our breast cancer cohort CA 15-3 was not recorded, and CEA was measured in only 19 patients, a statistical comparison was not possible. Studies which implicate these markers are planned in the future.

## Conclusion

In conclusion, based on the significant association between DNA and nucleosomes, DNA may predominantly circulate in form of nucleosomes in human blood. Although serum levels of nucleosomes and DNA in breast cancer patients are significantly elevated when compared with healthy controls, they are not useful for cancer diagnosis, owing to the increase in blood of patients with benign breast tumors. Nevertheless, the quantification of circulating nucleic acids as a rapid, non-invasive and blood-based screening tool could be used for the early detection of breast tumor progression, but larger studies with follow-up blood samples are required to corroborate this conclusion. The increased activities of caspases 3 and 7 observed in blood of the patients with advanced tumor stages and metastatic disease may contribute to the rise in nucleic acid concentrations in blood of cancer patients, but they do not seem to be relevant for the high serum levels of nucleic acids in patients with benign breast disease. Here, other caspases seem to be responsible for this release. The striking upregulation of the protease activities in patients with advanced tumor stages and metastatic disease reflects their role in tumor progression and metastases.

Considering the small sample sizes of our cohorts and the broad range of our data, additional studies to these preliminary results are warranted to obtain further information on the tumor activity and to determine the clinical relevance of the circulating nucleic acids in blood of breast cancer patients.

## Abbreviations

M0: patients with primary breast cancer; M1: patients with metastatic breast cancer; MMPs: matrix metalloproteinases; TIMPs: tissue inhibitors of metalloproteinases

## Competing interests

The authors declare that they have no competing interests.

## Authors' contributions

CR and HS performed all experiments. HS and CR performed the statistical analysis. HS drafted the manuscript and KP revised the manuscript. BR, VM, SKB and WJ prepared the clinical material. CR summarized the clinical parameters. HS and CR were involved in conception and design of the study and participated in the discussion and interpretation of the results. All authors read and approved the final manuscript.

## Pre-publication history

The pre-publication history for this paper can be accessed here:

http://www.biomedcentral.com/1471-2407/11/4/prepub

## References

[B1] Coughlin SS Ekwueme DU Breast cancer as a global health concern Cancer Epidemiol 2009 33 5 315 318 10.1016/j.canep.2009.10.003 19896917

[B2] Vetto JT Luoh SW Naik A Breast cancer in premenopausal women Curr Probl Surg 2009 46 12 944 1004 10.1067/j.cpsurg.2009.07.002 19887229

[B3] Fleischhacker M Schmidt B Circulating nucleic acids (CNAs) and cancer--a survey Biochim Biophys Acta 2007 1775 1 181 232 17137717 10.1016/j.bbcan.2006.10.001

[B4] Stroun M Lyautey J Lederrey C Olson-Sand A Anker P About the possible origin and mechanism of circulating DNA apoptosis and active DNA release Clin Chim Acta 2001 313 1-2 139 142 10.1016/S0009-8981(01)00665-9 11694251

[B5] Enari M Sakahira H Yokoyama H Okawa K Iwamatsu A Nagata S A caspase-activated DNase that degrades DNA during apoptosis, and its inhibitor ICAD Nature 1998 391 6662 43 50 10.1038/34112 9422506

[B6] Choi JJ Reich CF Pisetsky DS The role of macrophages in the in vitro generation of extracellular DNA from apoptotic and necrotic cells Immunology 2005 115 1 55 62 10.1111/j.1365-2567.2005.02130.x 15819697 PMC1782131

[B7] Rumore P Muralidhar B Lin M Lai C Steinman CR Haemodialysis as a model for studying endogenous plasma DNA: oligonucleosome-like structure and clearance Clin Exp Immunol 1992 90 1 56 62 10.1111/j.1365-2249.1992.tb05831.x 1395101 PMC1554541

[B8] Schwarzenbach H Kemper B Beeger C Otterbach F Kimmig R Pantel K S K-B Evaluation of cell-free tumor DNA in blood and disseminated tumor cells in bone marrow of patients with primary breast cancer Breast Cancer Res 2009 in press 19772563 10.1186/bcr2404 PMC2790848

[B9] Schwarzenbach H Muller V Beeger C Gottberg M Stahmann N Pantel K A critical evaluation of loss of heterozygosity detected in tumor tissues, blood serum and bone marrow plasma from patients with breast cancer Breast Cancer Res 2007 9 5 R66 10.1186/bcr1772 17915011 PMC2242661

[B10] Schwarzenbach H Muller V Stahmann N Pantel K Detection and characterization of circulating microsatellite-DNA in blood of patients with breast cancer Ann N Y Acad Sci 2004 1022 25 32 10.1196/annals.1318.005 15251935

[B11] Schwarzenbach H Pantel K Kemper B Beeger C Otterbach F Kimmig R Kasimir-Bauer S Comparative evaluation of cell-free tumor DNA in blood and disseminated tumor cells in bone marrow of patients with primary breast cancer Breast Cancer Res 2009 11 5 R71 10.1186/bcr2404 19772563 PMC2790848

[B12] Holdenrieder S Nagel D Schalhorn A Heinemann V Wilkowski R von Pawel J Raith H Feldmann K Kremer AE Muller S Clinical relevance of circulating nucleosomes in cancer Ann N Y Acad Sci 2008 1137 180 189 10.1196/annals.1448.012 18837945

[B13] Deligezer U Akisik EE Akisik EE Kovancilar M Bugra D Erten N Holdenrieder S Dalay N H3K9me3/H4K20me3 Ratio in Circulating Nucleosomes as Potential Biomarker for Colorectal Cancer Adv Exp Med Biol Ed Gahan P Springer 2010 97 104

[B14] Holdenrieder S Spuler A Tischinger M Nagel D Stieber P Presence of Nucleosomes in Cerebrospinal Fluid of Glioblastoma Patients - Potential for Therapy Monitoring Adv Exp Med Biol Ed Gahan P Springer 2010 79 84

[B15] Kohles N Nagel D Jüngst D Durner J Stieber P Holdenrieder S The Course of Circulating Nucleosomes in Liver Cancer Patients Undergoing Transarterial Chemoembolization Therapy Adv Exp Med Biol Ed Gahan P Springer 2010 73 78

[B16] Stoetzer OJ Fersching DMI Holdenrieder S Circulating Nucleosomes and DNAse in Breast Cancer Patients During Neoadjuvant Chemotherapy Adv Exp Med Biol Ed Gahan P Springer 2010 85 90

[B17] Kuroi K Tanaka C Toi M Plasma Nucleosome Levels in Node-Negative Breast Cancer Patients Breast Cancer 1999 6 4 361 364 10.1007/BF02966454 11091744

[B18] Kuroi K Tanaka C Toi M Clinical significance of plasma nucleosome levels in cancer patients Int J Oncol 2001 19 1 143 148 11408935 10.3892/ijo.19.1.143

[B19] Lopez-Otin C Matrisian LM Emerging roles of proteases in tumour suppression Nat Rev Cancer 2007 7 10 800 808 10.1038/nrc2228 17851543

[B20] Turk B Targeting proteases: successes, failures and future prospects Nat Rev Drug Discov 2006 5 9 785 799 10.1038/nrd2092 16955069

[B21] Soini Y Paakko P Lehto VP Histopathological evaluation of apoptosis in cancer Am J Pathol 1998 153 4 1041 1053 9777936 10.1016/S0002-9440(10)65649-0 PMC1853067

[B22] Vakkala M Paakko P Soini Y Expression of caspases 3, 6 and 8 is increased in parallel with apoptosis and histological aggressiveness of the breast lesion Br J Cancer 1999 81 4 592 599 10.1038/sj.bjc.6690735 10574243 PMC2362889

[B23] Deligezer U Erten N Akisik EE Dalay N Circulating fragmented nucleosomal DNA and caspase-3 mRNA in patients with lymphoma and myeloma Exp Mol Pathol 2006 80 1 72 76 15961076 10.1016/j.yexmp.2005.05.001

[B24] Friedl P Wolf K Tube travel: the role of proteases in individual and collective cancer cell invasion Cancer Res 2008 68 18 7247 7249 10.1158/0008-5472.CAN-08-0784 18794108

[B25] Aimes RT Zijlstra A Hooper JD Ogbourne SM Sit ML Fuchs S Gotley DC Quigley JP Antalis TM Endothelial cell serine proteases expressed during vascular morphogenesis and angiogenesis Thromb Haemost 2003 89 3 561 572 12624642

[B26] Pantel K Alix-Panabieres C Riethdorf S Cancer micrometastases Nat Rev Clin Oncol 2009 6 6 339 351 10.1038/nrclinonc.2009.44 19399023

[B27] Pantel K Brakenhoff RH Dissecting the metastatic cascade Nat Rev Cancer 2004 4 6 448 456 10.1038/nrc1370 15170447

[B28] Schwarzenbach H Alix-Panabieres C Muller I Letang N Vendrell JP Rebillard X Pantel K Cell-free Tumor DNA in Blood Plasma As a Marker for Circulating Tumor Cells in Prostate Cancer Clin Cancer Res 2009 15 3 1032 1038 10.1158/1078-0432.CCR-08-1910 19188176

[B29] Clark IM Swingler TE Sampieri CL Edwards DR The regulation of matrix metalloproteinases and their inhibitors Int J Biochem Cell Biol 2008 40 6-7 1362 1378 10.1016/j.biocel.2007.12.006 18258475

[B30] Hemsen A Riethdorf L Brunner N Berger J Ebel S Thomssen C Janicke F Pantel K Comparative evaluation of urokinase-type plasminogen activator receptor expression in primary breast carcinomas and on metastatic tumor cells Int J Cancer 2003 107 6 903 909 10.1002/ijc.11488 14601049

[B31] Wu ZS Wu Q Yang JH Wang HQ Ding XD Yang F Xu XC Prognostic significance of MMP-9 and TIMP-1 serum and tissue expression in breast cancer Int J Cancer 2008 122 9 2050 2056 10.1002/ijc.23337 18172859

[B32] Jiang N Pisetsky DS The effect of inflammation on the generation of plasma DNA from dead and dying cells in the peritoneum J Leukoc Biol 2005 77 3 296 302 10.1189/jlb.0704411 15601668

[B33] Zanetti-Dallenbach RA Schmid S Wight E Holzgreve W Ladewing A Hahn S Zhong XY Levels of circulating cell-free serum DNA in benign and malignant breast lesions Int J Biol Markers 2007 22 2 95 99 17549664 10.1177/172460080702200202

[B34] Schubbert R Lettmann C Doerfler W Ingested foreign (phage M13) DNA survives transiently in the gastrointestinal tract and enters the bloodstream of mice Mol Gen Genet 1994 242 5 495 504 10.1007/BF00285273 8121408

[B35] Holdenrieder S von Pawel J Dankelmann E Duell T Faderl B Markus A Siakavara M Wagner H Feldmann K Hoffmann H Nucleosomes, ProGRP, NSE, CYFRA 21-1, and CEA in monitoring first-line chemotherapy of small cell lung cancer Clin Cancer Res 2008 14 23 7813 7821 10.1158/1078-0432.CCR-08-0678 19047109

[B36] Kim MJ Park BW Lim JB Kim HS Kwak JY Kim SJ Park SH Sohn YM Moon HJ Kim EK Axillary lymph node metastasis: CA-15-3 and carcinoembryonic antigen concentrations in fine-needle aspirates for preoperative diagnosis in patients with breast cancer Radiology 2010 254 3 691 697 10.1148/radiol.09091031 20123899

[B37] Zeiwar MM Zaki SM Mohammad LA Zidan AA El Nagar MR HER-2 gene amplification, serum nucleosomes, CEA and CA15.3 tumor markers in breast cancer patients Egypt J Immunol 2007 14 2 29 41 20306655

